# Analyzing Nitrogen Effects on Rice Panicle Development by Panicle Detection and Time-Series Tracking

**DOI:** 10.34133/plantphenomics.0048

**Published:** 2023-06-23

**Authors:** Qinyang Zhou, Wei Guo, Na Chen, Ze Wang, Ganghua Li, Yanfeng Ding, Seishi Ninomiya, Yue Mu

**Affiliations:** ^1^ College of Agriculture, Academy for Advanced Interdisciplinary Studies, Collaborative Innovation Center for Modern Crop Production co-sponsored by Province and Ministry, Nanjing Agricultural University, Nanjing 210095, China.; ^2^ Graduate School of Agricultural and Life Sciences, The University of Tokyo, 1-1-1 Midori-cho, Nishi-Tokyo, Tokyo 188-0002, Japan.

## Abstract

Detailed observation of the phenotypic changes in rice panicle substantially helps us to understand the yield formation. In recent studies, phenotyping of rice panicles during the heading–flowering stage still lacks comprehensive analysis, especially of panicle development under different nitrogen treatments. In this work, we proposed a pipeline to automatically acquire the detailed panicle traits based on time-series images by using the YOLO v5, ResNet50, and DeepSORT models. Combined with field observation data, the proposed method was used to test whether it has an ability to identify subtle differences in panicle developments under different nitrogen treatments. The result shows that panicle counting throughout the heading–flowering stage achieved high accuracy (*R*^2^ = 0.96 and RMSE = 1.73), and heading date was estimated with an absolute error of 0.25 days. In addition, by identical panicle tracking based on the time-series images, we analyzed detailed flowering phenotypic changes of a single panicle, such as flowering duration and individual panicle flowering time. For rice population, with an increase in the nitrogen application: panicle number increased, heading date changed little, but the duration was slightly extended; cumulative flowering panicle number increased, rice flowering initiation date arrived earlier while the ending date was later; thus, the flowering duration became longer. For a single panicle, identical panicle tracking revealed that higher nitrogen application led to earlier flowering initiation date, significantly longer flowering days, and significantly longer total duration from vigorous flowering beginning to the end (total DBE). However, the vigorous flowering beginning time showed no significant differences and there was a slight decrease in daily DBE.

## Introduction

In today’s world, food security is one of the major issues faced by the world population. Rice is the third-largest food crop in the world. It is the main source of energy for nearly half of the world's population [1]. As a result, rice production plays an important role in ensuring global food security and protecting the health of human beings. The rice panicle is an essential reproductive organ of rice and influences the yield [2]. Therefore, the research regarding the panicle region is crucial for analyzing the growth status and yield of rice crop. In addition, the heading–flowering stage of rice crop is important during the reproductive cycle. During this stage, the rice crop is sensitive to external environmental factors, such as temperature and moisture, which ultimately affect the yield [3]. The phenotypes at this stage, such as panicle number, heading date, and flowering time traits, not only reflect the growth and development status of rice, but also affect the grain filling stage [4]. Using the field phenotype monitoring system to observe the rice panicles for understanding the dynamic changes in phenotypes, such as rice panicle morphological characteristics and panicle number in time series, is of great significance for crop cultivation, breeding, and plant physiology. For instance, it is well-known that the quantity of nitrogen affects panicle development [5], which, in turn, influences the yield. Therefore, a field phenotype monitoring system will promote the progress of cultivation technology.

However, it is noteworthy that in the present rice cultivation and breeding studies, such vital phenotyping information on rice panicles still largely relies on visual observations and manual measurements. This process is time-consuming, laborious, and inefficient. In addition, in large-scale field surveys, it is extremely difficult to obtain the data due to the subjectivity and the similarity between individual plants. It is also difficult to observe a large number of field plots simultaneously or with high frequency along with a time series. Moreover, tracking the growth and development of individual rice panicles and understanding the precise changes in flowering time, grain filling initiation, and other important information assist us in analyzing the individual growth differences, which are difficult to achieve manually.

Recently, computer vision and machine learning have been extensively used in various fields. The deep neural networks have effectively addressed various tasks, such as image classification, object detection, and semantic segmentation, and achieved excellent results [6]. As a field of machine learning, deep learning is driven by large and dynamic datasets. However, it does not require domain experts to set the target data features and could automatically extract high-dimensional features. Deep learning has been successfully applied in the field of plant phenotyping, such as variety identification [7], stress response identification [8], fruit detection [9,10], leaf counting [11], rice panicle segmentation [12], and weed segmentation based on pixel segmentation [13].

Guo et al. [14] used the scale-invariant feature transform (SIFT) algorithm to extract the feature points from the Red-Green-Blue (RGB) images of rice spikelet and then used the support vector machine to detect the flowering panicles of rice. However, if different initial centroids are selected in the SIFT algorithm, different features appear in the same image. Xiong et al. [12] developed a rice panicle segmentation method, namely, Panicle-SEG, based on deep learning and super-pixel optimization by simulating natural conditions and generating rice panicle candidate regions, and realized the counting of panicles. However, this approach typically requires large datasets with object-level annotations, which can be laborious for tasks that only require quantitative calculations. Desai et al. [15] used a convolutional neural network (CNN) for classifying the flowering stage of rice panicles and predicted the heading date of rice by calculating the detected flowering area, with an absolute error of less than 1 day. Yang et al. [16] used FPN-Mask to segment the panicles and leaves from the heading stage to the mature stage, achieving pixel accuracies of 0.99 and 0.98 for panicles and leaves, respectively. The authors also proposed a new index called leaf-to-panicle ratio (LPR) and compared the effect of nitrogen application during different stages of growth. Wang et al. [2] recently proposed a method for removing duplicate detection and demonstrated that their method outperforms the existing non-maximum suppression (NMS) method, which is specifically designed for detecting rice panicles in field images, with large image sizes. The mean absolute percentage error and accuracy of this YOLO v5-based model were 3.44% and 92.77%, respectively.

Although the aforementioned studies effectively extract the phenotypic characteristics of rice panicles, their performance is limited for the dynamic growth and development of rice panicles. The target tracking method can integrate information of time series for achieving more detailed phenotyping. The visual object tracking is one of the most elementary human visual functions. It is also one of the most important research directions in the field of machine vision [17]. As compared to target detection, the target tracking tasks not only make up for the missed and false detections in target detection but also result in a smoother output and achieve continuous target frame annotation. Based on the class number of target objects being tracked, the target tracking methods can be divided into single-target tracking and multi-target tracking [18]. According to the change of perspective, it can be divided into single-view target tracking and multi-view target tracking. This work utilizes a field-fixed camera platform [19] for acquiring data in the form of a single view, and then performs single-class multi-object tracking. By tracking the changes in multiple rice panicles in the time-series images, a continuous output could be obtained. This enables us to obtain more detailed information, such as changes in flowering time, duration of flowering, grain filling dynamics, and other phenotypic information.

Currently, there are many target tracking methods based on deep learning. In the hierarchical convolutional tracking method proposed by Chao et al [20], the visual representation of the target object is established, followed by the construction and optimization of discriminative correlation filters. Danelljan et al. first used a pre-trained CNN model for extracting the visual representation of the target object. Then, the hierarchical visual representation is fused in a continuous topological space, and the Continuous Convolution Operators for visual Tracking algorithm [21] and the optimized Efficient Convolution Operators algorithm [22] are proposed. Simple Online and Realtime Tracking (SORT) [23] uses Faster R-CNN [24] to predict and track the target motion trajectory based on Kalman filter and then uses the Hungarian binary matching [25] algorithm to associate the detections and tracking boxes. The DeepSORT [26] algorithm is designed based on the SORT algorithm by adding cascade matching, depth appearance features, and person re-identification (ReID) network extraction as the embedding layer; adding Mahalanobis distance and cosine distance metrics; solving the partial occlusion problem; and reducing the number of ID switching. The algorithm achieves a relatively stable tracking effect and is widely used in the tracking and detection of industrial targets, such as vehicles and pedestrians [27,28].

In the present rice cultivation and breeding studies during the heading–flowering stage, phenotyping of rice panicles still lacks comprehensive analysis, especially of rice panicle development such as dynamic changes analysis of heading/flowering panicle number and single panicle flowering time. In addition, monitoring and analyzing the effects of nitrogen on panicle development during the heading–flowering stage may help us make more appropriate cultivation and management decisions. This work aims to:

1. Build a pipeline for estimating the rice panicle number, heading date, panicle flowering time, and flowering time duration based on deep learning and time-series images, by using the YOLO v5 (https://github.com/ultralytics/yolov5), ResNet 50 [29], and DeepSORT models.

2. Test whether the pipeline has the ability to identify the changes of agronomically important trait, such as slightly different responses of panicle development under different nitrogen treatments.

## Materials and Methods

### Field experiment design

The experiment was set up in Danyang, Jiangsu Province, China (31°54′28.91′′N, 119°28'9.65′′E). Two local common varieties were planted in 2019 and 2020. The size of the field plot was 3.3 m × 2.6 m, with rice planted in 30-cm row spacing and 18-cm column spacing. In 2019, one field plot was set and the nitrogen fertilization was 150 kg hm^−2^ (N150). In 2020, 3 field plots were set with different nitrogen treatments, i.e., 300 kg hm^−2^ (N300), 270 kg hm^−2^ (N270), and 240 kg hm^−2^ (N240). Please note that the urea was used as the sole nitrogen fertilizer. Each plot of the experimental field was strictly isolated to prevent the flow of water and fertilizer between different nitrogen fertilizer plots.

### Data acquisition and dataset preparation

**Fig. 1.**
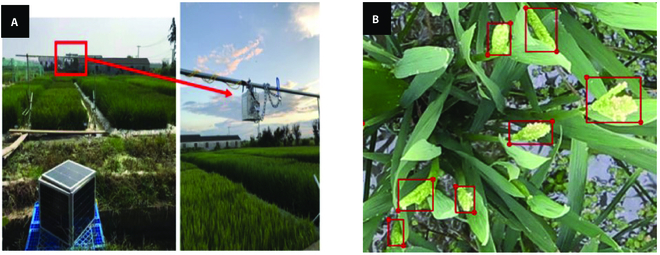
Image acquisition system (A) and annotation examples (B). The red box in (A) shows the position of the camera above the experimental paddy field. The red boxes in (B) represent the examples of the annotated rice panicles.

In this experiment, a solar energy-based automatic field fixed crop image acquisition and transmission device [19] was used to acquire the ground images, as presented in Fig. 1A. The camera used for image acquisition is Canon EOS M5 (22.3 × 14.9 mm full-pixel dual-core complementary metal oxide semiconductor, CMOS), with a resolution of 6,000 × 4,000 pixels. We used Raspberry Pi and Gphoto2 software to acquire the images automatically. The image acquisition device was installed on a fixed platform built in the target field. The camera shot vertically downwards at an angle of 90°. The height of the camera from the ground was about 3 m, the image resolution on the ground was about 71 pixels/cm, the size of the coverage area was about 1.2 m × 1.4 m, and the camera shooting interval was set to 5 min from 8:00 to 18:00 during the heading–flowering stage. The images were directly uploaded to a cloud server in real time. In order to unify the timeline, we converted the date into number of days after transplanting and recorded it as the day after transplant (DAT). We built 4 datasets for panicle detection, panicle heading and flowering phenotyping analysis, and individual panicle tracking as presented in Table 1.

**Table 1.** Summary of the datasets used in this study. The numbers in dataset_1, dataset_3, dataset_4 are the numbers of sub-images, while the number in dataset_2 represents the number of cropped single panicle images.

Dataset ID	Time	Datasets	Year		Total
			2019	2020	
Dataset_1	DAT 59-DAT 68	Training	768	332	1,100
		Validation	192	83	275
Dataset_2	DAT 59-DAT 68	Training	1,098	2,166	3,264
		Validation	218	598	816
Dataset_3	DAT 58-DAT 71		126	378	504
Dataset_4	DAT 58-DAT 71		574	1,722	2,296

First, we randomly selected 10 images per day from the daily time-series images between DAT 59 and DAT 68 in the 2019 image dataset (100 original images were selected). These images completely covered the whole heading–flowering stage. Then, we divided the original image evenly into 16 sub-images, each with a size of 1,500 × 1,000 pixels and no overlap, and used them to build the initial rice panicle detection model. In this work, the LabelMe [30] tool was used for data annotation. The minimum resolution of rice panicles should not be less than 20 × 20 pixels; otherwise, they were ignored. The annotation results included information of the frames, such as image name, label name, and the location of the target frame. Figure 1B presents an example of data labeling, where the red boxes represent the annotated rice panicles.

In order to enhance the applicability of the initial model, we randomly added 2 images per day of each plot under 3 nitrogen treatments between DAT 59 and DAT 68 in the 2020 image dataset (60 original images) to the 100 images selected in 2019 image dataset. Then, we used the same way to annotate the panicles. The training and validation datasets of panicle detection model were defined as dataset_1. This dataset comprised images acquired at different times, for different nitrogen treatments, and in different light environments. In order to identify the flowering panicles, we used the detections obtained by the detection model, and manually divided them into 2 categories based on visual perception by experts with agronomy background, i.e., vigorous flowering and non-vigorous flowering. Thus, more than 4,000 detected single panicle images were cropped from images in dataset_1, which constitute dataset_2, as shown in Table 1.

To study the heading–flowering stage phenotyping, one image was randomly selected every hour from 8:00 to 16:00 (9 original images) during DAT 58 to DAT 71 (14 days), including images collected in 2019 and 2020 (4 treatments). We cropped the central part of each original image, resulting in a total of 504 sub-images with a size of 1,500 × 1,000 pixels (dataset_3). Then, dataset_4 was built based on dataset_3 by adding more images every 15 min during 8:00 to 10:00 and 14:00 to 16:00, and every 10 min during 10:00 to 14:00, considering the computational load for the detection process. Finally, we had 41 images every day, and a total of 2,296 sub-images (dataset_4) for performing detailed analysis of the flowering process.

### Pipeline for monitoring the panicle development

**Fig. 2.**
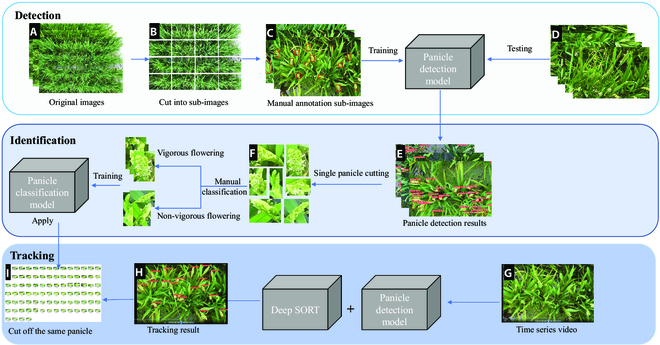
The flowchart of panicle detection, flowering panicle identification, and individual panicle tracking. (A) Original high-resolution image. (B) Cutting into sub-images. (C) Manual annotation. (D) Panicle detection model generation and testing. (E) Detection results sample. (F) Crop the panicles from the detection results. (G) Integrate images into a video. (H) Panicle tracking based on panicle detect model and DeepSORT. (I) Apply the classification model to analyze the tracking results.

The workflow of the proposed pipeline is presented in Fig. 2. For panicle detection, YOLO v5 was chosen as the panicle detection model. The panicle detection comprised 4 steps: (a) The original images were divided evenly into 16 sub-images, as presented in Fig. 2B. (b) The sub-images were manually annotated using LabelMe, as presented in Fig. 2C. (c) The panicle detection model was trained by the annotated sub-images. (d) The detection model was evaluated, as presented in Fig. 2D.

For the flowering panicle identification, we used ResNet50 to classify the vigorous flowering panicles and non-vigorous flowering panicles. This process also comprised 4 steps: (a) All the single panicles were cropped using the detection results, as presented in Fig. 2E. (b) The panicles were manually divided into 2 categories, as presented in Fig. 2F. (c) The panicle classification model was trained. (d) The classification model was evaluated.

For identical panicle tracking, DeepSORT and YOLOv5 models were used for tracking one single panicle. This process comprised 3 steps: (a) Time-series images were integrated into video, as presented in Fig. 2G. (b) The panicle detection model, as previously trained, was used, and the pre-trained ReID model was used to track the identical panicle, as presented in Fig. 2H. (c) The same panicles were cropped using the tracking results and the panicle classification model was used for flowering analysis, as presented in Fig. 2I.

### Building the model for monitoring panicle development

In this work, 1,375 sub-images were obtained for training and evaluating the panicle detection model. Then, we randomly selected 1,100 sub-images for training (dataset_1) and 275 sub-images for validation (dataset_1), in a ratio of 8:2. The panicle detection model was built based on YOLO v5s6. The model training parameters included learning rate = 0.001, momentum = 0.937, decay = 0.0005, batch size = 16, confidence threshold = 0.25, and epochs = 100. The mini-batch gradient descent was used to optimize the training model. We also used the deep neural network acceleration libraries in this work. The network was pre-trained on the COCO128 dataset based on the COCO-Train2017 dataset on Microsoft COCO [31], and the resulting weights were used as the initial weights.

For training the panicle classification model, dataset_2 was used as the input. This dataset comprised the cropped single panicle images detected by the panicle detection model built in the last step. Eighty percent of the images were used for training, and 20% were used for validation. The panicle classification model was trained using the transfer learning method based on ResNet50, which was pre-trained on PyTorch (https://pytorch.org/hub/nvidia_deeplearningexamples_resnet50/). The learning rate of the model was 0.0001, the learning rate decay mode was fixed, the batch size was 8, and the model was trained for 150 epochs.

For the identical panicle tracking, dataset_4 was used. This dataset comprised 41 images per day, which were obtained from video frames. They were used to track the panicles of 2019 and 2020 by combining the panicle detection model with DeepSORT. This enables us to realize the tracking of rice panicles during a single day. Some tracking results are presented in supplementary videos (Supplementary Material 1). Max-distance, min-confidence, nms-max-overlap, max-age, max-IOU-distance, and other evaluation parameters were used to filter the output results.

This work ran on one computer with the Ubuntu 16.04 system, Inter Xeon E5 CPU (2.20 GHz per CPU core, 32 GB of memory), and NVIDIA Tesla V100-DGXS GPU with 16 GB memory.

### Evaluation of the panicle detection model, the panicle classification model, and heading date estimation

In order to evaluate the effectiveness of the panicle detection model, the validation dataset in dataset_1 was used for calculating the detecting accuracy and counting accuracy. The detection accuracy was measured by precision, recall, F1-score, and mean average precision when Intersection over Union (IoU) ≥ 0.5 (mAP_50_). As we know, precision and recall are mutually restricted in practice, and the single comparison will be ambiguous. Therefore, we used the mAP coefficient from the official COCO repository (https://github.com/cocodataset/cocoapi/tree/master/PythonAPI), which is one of the most important metrics to evaluate the performance of target detection algorithms. The indicators were evaluated using the following formulas:

$$\mathrm{Precision}=\mathrm{TP}/\left(\mathrm{TP}+\mathrm{FP}\right)$$ (1)

$$\mathrm{Recall}=\mathrm{TP}/\left(\mathrm{TP}+\mathrm{FN}\right)$$ (2)

$$F1-\mathrm{score}=\left(2\times \mathrm{Precision}\times \mathrm{Recall}\right)/\left(\mathrm{Precision}+\mathrm{Recall}\right)$$ (3)

where TP, FP, and FN denote the number of true positives, false positives, and false negatives, respectively.

The coefficient of determination (*R*^2^) and root mean square error (RMSE) were determined as evaluation indicators to measure the counting performance of the proposed model. The indicators were evaluated using the following formulas:

$$R^{2}=1-\frac{\sum_{i=1}^{n}{\left(c_{i}-{\hat{c}}_{i}\right)}^{2}}{\sum_{i=1}^{n}{\left(c_{i}-\bar{c}\right)}^{2}}$$ (5)

$$\mathrm{RMSE}=\sqrt{\frac{1}{n}\Sigma_{i=1}^{n}{\left(c_{i}-{\hat{c}}_{i}\right)}^{2}}$$ (6)

where *n* represents the number of rice panicle images, while *c_i_* and ${\hat{c}}_{i}$ represent the number of rice panicles labeled and estimated by the model in the *i*th image, respectively, and $\bar{c}$ represents the average number of labeled rice panicles.

In order to evaluate the effectiveness of panicle classification model, we used the validation dataset in dataset_2 for computing the precision, recall, and accuracy. The accuracy is mathematically expressed as follows:

$$\mathrm{Accuracy}=\left(\mathrm{TP}+\mathrm{TN}\right)/\left(\mathrm{TP}+\mathrm{TN}+\mathrm{FP}+\mathrm{FN}\right)$$ (7)

where TN denotes the number of true negatives.

For heading date estimation, dataset_3 was used for visually performing observations and evaluations.

In this research, the value of panicle number (pn) was defined as the average of panicles each day based on the cropped sub-images in dataset_3. The number of manually counted panicles in each image was regarded as the ground truth and the heading date is then calculated.

## Results

### Model performance evaluation

#### Panicle detection model evaluation

**Fig. 3.**
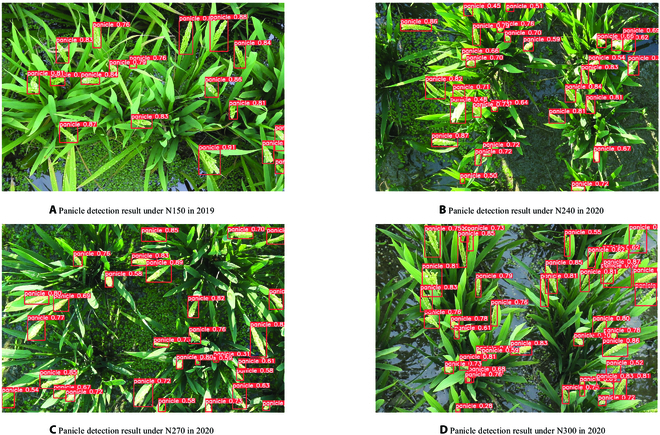
Examples of panicle detection results of images captured in 2019 (A) and 2020 (B to D) under different nitrogen treatments. The red boxes in the figure are the detected rice panicles, each box corresponds to one panicle, and the number represents the panicle confidence.

**Fig. 4.**
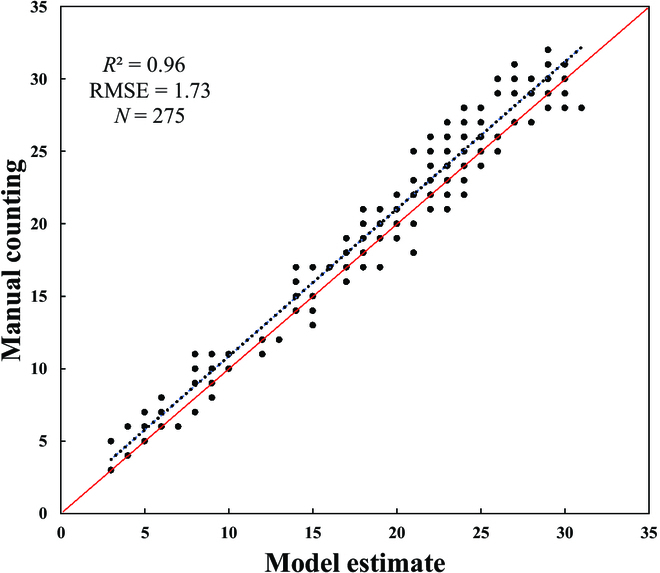
Scatter plot of manual counting and model estimate of rice panicle number using the validation dataset in dataset_1.

For panicle detection and counting, model training and validation were performed using the training and validation datasets presented in Table 1. The training results and label information presented in Fig. S1 (Supplementary Material 1) show that the YOLO v5 model does not suffer from overfitting, underfitting, or gradient disappearance. As shown in Fig. 3, the panicle detection model is effective for rice panicle detection of different nitrogen treatments, even when the shape, color, and texture of the rice panicles are different. In addition, as presented in Table 2, accuracy of panicle detection in 2 years is relatively similar, which indicates that the universality of the model for different rice varieties is also ideal. Moreover, the panicle detection model was evaluated by comparing the error between manual counting and the count estimated by the model. The correlation analysis is presented in Fig. 4. The *R*^2^ and RMSE for the detection model is 0.96 and 1.73, respectively. Figure 4 shows that more than half of the samples are above the 1:1 line, indicating that the model tends to be slightly underestimated. The results shown in Table 2 and Fig. 4 indicate that the model obtains a relatively high accuracy in detecting rice panicles, and the change of detected panicle number with time will be helpful for the researchers to identify the heading date.

**Table 2.** Accuracy of panicle detection for 2 years.

	Images	F1-score	Precision	Recall	mAP_50_
2019	192	0.877	0.913	0.844	0.892
2020	83	0.891	0.882	0.901	0.901
Total	275	0.882	0.904	0.861	0.895

#### Panicle classification evaluation

**Fig. 5.**
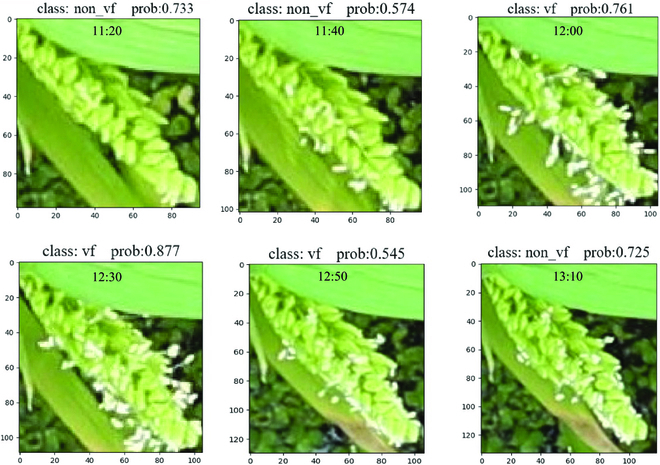
Examples of the identification of vigorous flowering panicles (vf) and non-vigorous flowering panicles (non_vf) from time-series images. The prob value represents the confidence of the corresponding class.

In order to classify flowering panicles, the panicle classification model was built based on ResNet 50, and achieved precision, recall, and accuracy values of 0.934, 0.975, and 0.955, respectively. Through this model, the vigorous flowering panicles (vf) and non-vigorous flowering panicles (non_vf) were identified. Examples of flowering panicle identification results are presented in Fig. 5. The change of probability values with time (Fig. 5) demonstrates that the flowering status of the panicle is efficiently identified on different times, and the flowering identification is in line with common sense. The results indicate that the model obtains a relatively high accuracy in flowering panicle classification, and the change of vigorous flowering panicle number with time can enable the researchers to analyze the rice flowering process.

### Heading date identification

**Fig. 6.**
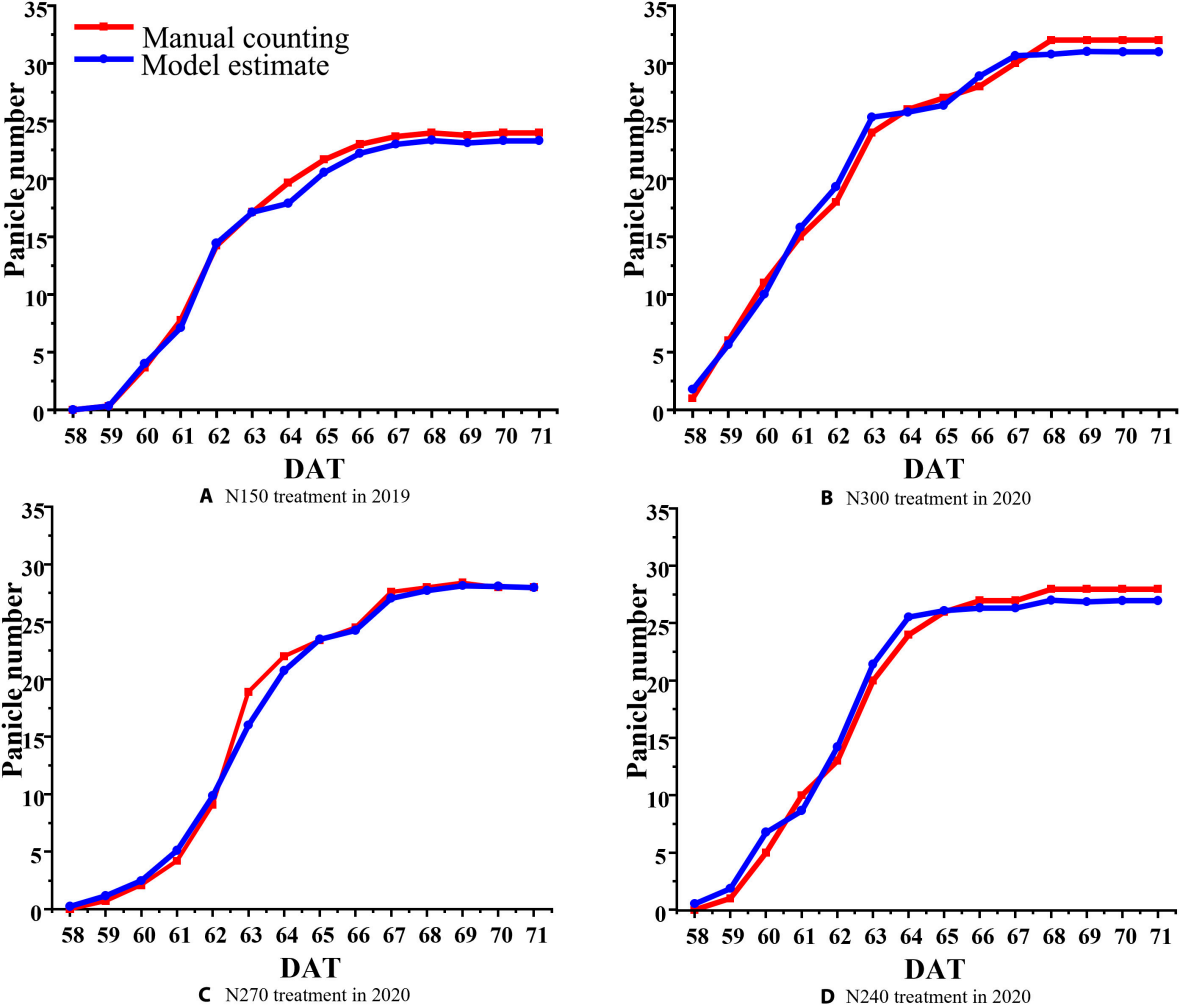
Cumulative distributions of average panicle numbers obtained by manual counting and model estimating under nitrogen treatment of N150 (A), N300 (B), N270 (C), and N240 (D). The average panicle numbers were calculated from the 9 images in each hour (8:00 to 16:00) every day.

The record of heading date is usually obtained by agronomists through field observations based on suitable plant samples in the field. This information is then used to determine the rice growth stages. The existing studies use 10%, 50%, and 80% of the stable heading panicle number (mean value of the numbers on the plateau in Fig. 6) to determine the initial heading date, the heading date, and the full heading date [32], respectively. Figure 6 shows the time-series changes of cumulative panicle number in the manual counting and the model estimate during the entire heading–flowering stage in 2019 and 2020. The value of pn was calculated as the average of detected panicles each day based on the sub-images in dataset_3. As presented in Fig. 6, the blue line and red line almost overlap, indicating that the model accurately estimates the growth of panicle number, regardless of the years, varieties, days, and nitrogen treatments. It implies that the heading date can be further determined.

**Fig. 7.**
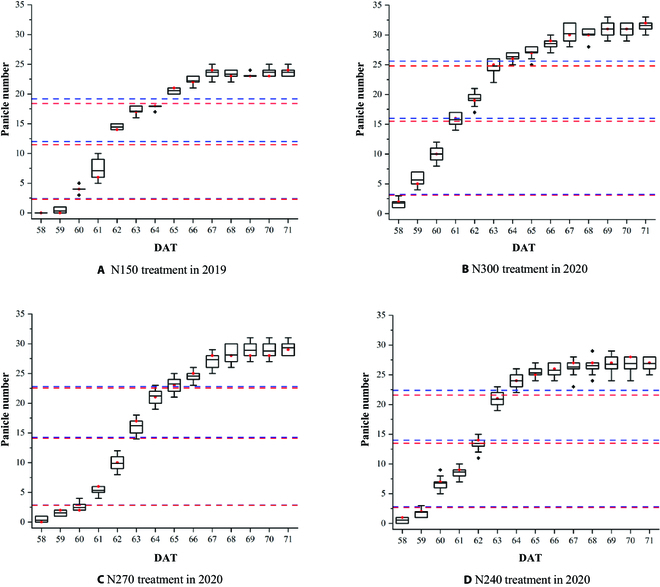
Comparison of cumulative panicle numbers and heading dates under different nitrogen treatments, i.e., N150 (A), N300 (B), N270 (C), and N240 (D). The red lines and the blue lines in each graph indicate the cumulative probabilities at 10%, 50%, and 80% of the stable panicle number by model estimate and manual counting, respectively. In each box plot, the black line and the red dot indicate the mean and the median, respectively.

In order to evaluate the heading date identification based on the panicle number estimated by our proposed model, we compared the cumulative panicle numbers of manual counting and model estimated at 10%, 50%, and 80% levels. The results are shown in Fig. 7. When both the median and the mean in the box plot exceed the dashed line, the first day was recorded as the corresponding heading date of the rice in the plot. Table 3 shows the comparison of heading date between manual observation and the estimation using the proposed method. It is evident from Table 3 that the error in the estimated heading date reaches the mean absolute error of 0.25 days for 4 treatments. The proposed model shows good performance under different nitrogen application levels. Moreover, the initial heading date and full heading date were also estimated. With an increase in the amount of nitrogen application in 2020, the 3 kinds of heading date have almost no difference among treatments, while the heading duration (from initial heading date to full heading date) becomes slightly longer (Table 3). In addition, the stable panicle number among treatments (Fig. 6) shows that the panicle number increases by increasing the nitrogen application.

**Table 3.** Comparison of heading dates after transplanting between the estimation using the proposed method and manual observation.

Dataset	Stable panicle number	Initial heading date (estimated)	Full heading date (estimated)	Heading date (observed)	Heading date (estimated)	Estimation error (days)
N150 2019	23.11	DAT 60	DAT 65	DAT 62	DAT 62	0
N300 2020	31.33	DAT 59	DAT 64	DAT 62	DAT 61	1
N270 2020	28.16	DAT 61	DAT 65	DAT 63	DAT 63	0
N240 2020	26.89	DAT 60	DAT 64	DAT 63	DAT 63	0
Mean absolute error (days)						0.25

### The change of flowering panicle number with time series

**Fig. 8.**
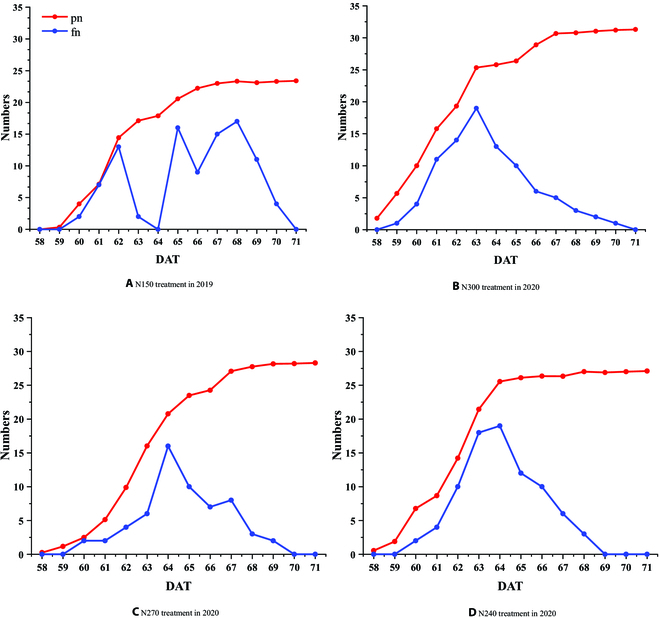
Comparisons of panicle number (pn, red line) and flowering panicle number (fn, blue line) during the heading–flowering stage under different nitrogen treatments, i.e., N150 (A), N300 (B), N270 (C), and N240 (D).

In order to understand the flowering process of rice populations during the flowering stage, the flowering rice panicles were classified into vigorous and non-vigorous flowering panicles. Then, the maximum number of detected vigorous flowering panicles was recorded as the flowering panicle number (fn) on that day (Fig. 8). It is evident from Fig. 8A that on DAT 63 and DAT 64, panicles almost did not flower. From the meteorological data of field climate station, it is known that DAT 63 was a cloudy and rainy day, with almost no sunlight and relatively low temperature. The lowest temperature was 20 °C and the humidity was high, and DAT 64 had rain all day long. The previous studies [33,34] show that low temperature and rain during the flowering period of rice seriously affect the flowering and pollination of rice. Consequently, that fact that no flowering occurred is reasonable. This result shows that the proposed method can accurately detect the sensitive flowering changes caused by the changes in the external environment. Figure 8B to D present the dynamic changes of rice panicle in plots under 3 different levels of nitrogen treatment. These 3 figures clearly demonstrate the flowering to decaying process of panicle development, which are almost consistent. This result shows that the proposed model has good adaptability under different nitrogen treatments.

By comparing the pn and fn curves, when the maximum flowering number of the group reaches the peak, the rice heading also enters a period of slow growth. The flowering duration is recorded from the day of the first panicle flowering to the last panicle flowering. This process generally lasts for 10 to 12 days. The flowering duration for the N300 treatment is DAT 59 to DAT 70, i.e., 12 days; that for the N270 treatment is DAT 60 to DAT 69, i.e., 10 days; and that for the N240 treatment is DAT 60 to DAT 68, i.e., 9 days. It shows that with an increase in the application of nitrogen fertilizer, the flowering starts earlier, and the flowering duration also becomes longer. In addition, the changes of flowering panicle number per 10 to 15 min on each day are shown in Fig. S2 (Supplementary Material 1). It is also consistent with our results mentioned above.

The vigorous flowering time of rice populations has also been estimated during each day. The moment when the flowering panicle numbers reach the peak was recorded as vigorous flowering time (VFT_g_) of the rice group on that day. In order to evaluate the proposed method, images were selected from DAT 62 to DAT 67. During these 6 days, the maximum flowering panicle number was larger than 5, and it was easy to judge if the flowering occurred or not by manual observation. This effectively reduces the human error, and is also in line with the actual situation. Table 4 shows the difference between the estimated VFT_g_ and manual observations from the images. The estimate of VFT_g_ is performed every day, with the error ranging from 0 to 20 min, and nearly half of the estimation errors are 0 min. The mean errors under the 3 different nitrogen treatments are 6.67 min, 8.33 min, and 6.67 min, respectively, with no significant difference. In addition, there is little difference in VFT_g_ between different nitrogen treatments within the same days. The rice flowering seems to depend more on the environmental conditions than on the nitrogen treatments.

**Table 4.** Vigorous flowering time of the rice group comparison between manual observations and model estimate under different nitrogen applications. Values followed by different parameters mean significantly different at *P* < 0.05 among different treatments.

DAT	N300			N270			N240		
	Manual	Estimated	Error (min)	Manual	Estimated	Error (min)	Manual	Estimated	Error (min)
62	11:00	11:10	10	11:10	11:20	10	11:00	11:10	0
63	11:40	11:40	0	11:40	12:00	20	11:40	11:40	0
64	11:50	11:50	0	12:00	12:00	0	11:50	11:40	10
65	11:50	11:50	0	11:30	11:40	10	11:40	11:50	10
66	12:00	11:40	20	12:00	11:50	10	11:40	12:00	20
67	11:30	11:40	10	11:40	11:40	0	11:40	11:40	0
Mean error (min) 6.67^a^					8.33^a^			6.67^a^		

**Fig. 9.**
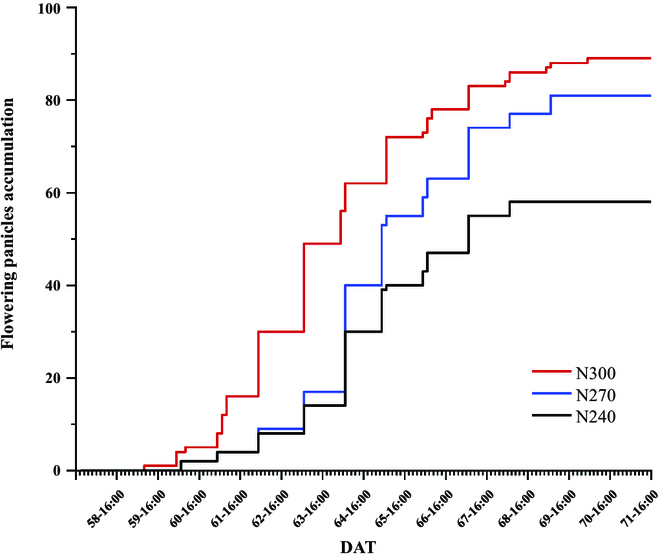
Cumulative distributions of flowering panicle number under different nitrogen treatments. It has a 1-h interval between each scale on the *x*-axis, a total of 9 scales from 8:00 to 16:00 each day.

Figure 9 shows the cumulative curve of flowering panicle number, which is calculated by cumulating fn values of each image in dataset_3 every day. It is evident from Fig. 9 that the larger the amount of nitrogen application, the larger is the cumulative total number of flowering panicles, and the earlier is the flower occurring. This means that the frequency of observed flowering is increased, which is due to effects in 2 aspects. On one hand, the increase in the panicle number leads to an increase in the flowering panicle accumulation number, which is reflected in Fig. 7 and Table 3. On the other hand, the longer the flowering duration is, the higher is the cumulative number. However, it is difficult to accurately analyze how these 2 aspects affect rice groups. Moreover, the cumulative curve has the problem of repeatedly counting the same flowering panicles. Therefore, we need to locate and track the individual panicles to discover more details in the flowering process.

### Identical panicle tracking in the heading–flowering stage

For identical panicle tracking, the panicle detection model (based on YOLO v5) combined with the DeepSORT model was applied on dataset_4, which comprised 41 images acquired per day arranged as video frames. Then, we tracked rice panicles every day. The information from the corresponding image was obtained, then the image of the target panicle was cropped from the original image and named based on the frame ID. In this way, all the images of individual target panicles within 1 day were obtained. Some tracking examples, i.e., tracking of rice panicles on DAT 67 under 4 nitrogen treatments, are presented in supplementary videos (Supplementary Material 2).

Based on the tracking results, the number of correctly tracked rice panicles that were continually tracked with the same IDs from the first frame to the last frame was counted. Then, the daily accuracy was calculated by the ratio of the correctly tracked rice panicle number divided by the panicle number in the first frame. The mean accuracies from DAT 62 to DAT 67 for N150, N300, N270, and N240 are 0.88, 0.72, 0.71, and 0.74, respectively. The tracking result is better for 2019 as compared to 2020, which is due to the more complex environment of 2020 data, i.e., more reflections from the water surface and more duckweeds. In terms of overall tracking results, about 70% of the panicles can be tracked continuously and completely. Due to abrupt changes in the external environment (such as wind or sunlight) and rice growth, the images in adjacent periods may change greatly and panicles may move suddenly and dramatically, making the tracking IDs of some panicles change. In order to reduce the errors caused by the changes in IDs during the tracking process, we integrate images of the same panicle with the aid of manual visual inspection.

Ten correctly tracked panicles were randomly selected for each nitrogen treatment, and the target panicle images were cropped. Then, the panicle classification model was used to determine the flowering initiation day after transplanting (FDAT), flowering days (FDs), vigorous flowering time of single panicle (VFT_p_) on each day, duration from vigorous flowering beginning to the end on each day (daily DBE), and the total of daily DBE on multi-days (total DBE). FDs was calculated as the sum of days when vigorous flowering occurs. VFT_p_ was defined as the moment that vigorous flowering starts continuously appearing on each day. For the convenience of comparison, we determined the VFT_p_ at the cumulated minutes from 8:00 on each day. Table 5 compares FDAT, FDs, VFT_p_, and total DBE under different nitrogen treatments. The results show that high application of nitrogen leads to an earlier FDAT, significantly longer FDs, and significantly longer total DBE. However, VFT_p_ shows no significant difference and there is a slight decrease in mean daily DBE.

**Table 5.** Flowering phenotype analysis in single panicle by tracking. Values followed by different parameters mean significantly different at *P* < 0.05 among different treatments.

Nitrogen treatment	Mean FDAT (DAT)	Mean FDs (days)	Mean VFT_p_ (min)	Mean total DBE (min)	Mean daily DBE (min)
N300	61.20^b^ ± 1.40	4.40^a^ ± 0.70	211.23^a^ ± 27.32	399.50^a^ ± 138.85	93.68^a^ ± 38.40
N270	62.60^a^ ± 1.51	3.90^ab^ ± 0.57	209.28^a^ ± 7.19	373.50^ab^ ± 127.30	94.28^a^ ± 23.80
N240	62.00^ab^ ± 1.33	3.60^b^ ± 0.52	202.08^a^ ± 13.97	348.00^b^ ± 107.27	95.92^a^ ± 22.52

FDAT, flowering initiation day after transplanting; FDs, flowering days; VFT_p_, vigorous flowering time of single panicle in each day; daily DBE, duration from vigorous flowering beginning to the end in each day; total DBE, total of daily DBE on multi-days.

## Discussion

### Panicle detection and counting

By calculating the precision, recall, F1-score, and average precision (Table 2), it is evident that the panicle detection model that is based on YOLO v5 performs well under different datasets during 2 different years. Compared with the research results presented by Zhou et al. [35] and Xu et al. [36], their detection precision reached 0.868 and 0.87, respectively, and our panicle detection model performs better (0.904). There is a slight drop in precision for the 2020 dataset as we increased the height of the camera a little bit (~0.2 m), thus reducing the size of the panicles in the original image. Secondly, as there were more duckweeds and weeds in the paddy field during the second year, it disturbs the panicle detection of the proposed model, although the YOLO v5 model is less affected by the background as compared to other deep learning models, such as the R-CNN series [37].

The difference between detected and manually counted panicle number is also calculated as shown in Figs. 4 and 6. The detection maintains high similarity with manual counting in terms of estimation error, coefficient of determination, and change curve with time series. This indicates that the number of panicles obtained using the model is reliable and accurate. However, when cropping the original image into sub-images for panicle detection, there is a risk of boundary box truncation, where the panicle is cut off at the edge of the image. It causes rice panicles to split into multiple pieces, which can adversely affect counting, classification [38], and tracking accuracy [39]. To address these issues, some researchers chose to merge the edge targets [9] or overlapped image edges while cutting. An improved NMS method has been proposed to handle repeated detections [2], as well as new modules such as Fitness NMS and Bounded IoU Loss [40]. In future studies, we will consider merging the edge targets as a post-processing optimization or using sub-images with overlap at the edges. We can also explore improved NMS algorithms or develop new duplicate detection reduction methods to eliminate overlapping targets and enhance accuracy.

In the actual field environment, high-density planting of rice results in denser and severe shading of panicles in the later stages of growth. On the other hand, different shooting angles also influence the detection of panicles. This is a common problem in the detection of panicles at present. Moreover, in fields with dense panicles, even experts have to perform calculations many times for obtaining the reliable measurement results. In order to further improve the effectiveness of panicle detection and counting, using different planting densities and increasing the number of acquisition angles in subsequent experiments may expand the adaptability of the model and improve its robustness.

As shown in Fig. 7 and Table 3, the proposed method is quite accurate in detecting panicles and identifying the heading date. In the existing studies, the investigation of heading date is hardly performed every hour. Thus, it is easy to overlook the change in the number of fast-growing rice panicles during the heading stage within 1 day. In this research, we calculated the daily mean and median values of panicle numbers by using the 9 images acquired by the hour during 8:00 to 16:00. This ensures that the estimated heading date is more reliable. In addition, during the period of rice rapid growth, we can see gaps in the box plot between 2 adjacent days in Fig. 7 (e.g., gap in the *y*-axis between DAT 61 and DAT 62 in Fig. 7A), which implies the growth process of rice in very early morning (before 8:00) or late afternoon (after 16:00) or even at night. In future studies, we can use longer observation time and other imaging methods to obtain the images at night, to analyze the rice growth circadian pattern, and to provide more useful information to researchers.

It is worth mentioning that the proposed method discovers the subtle changes of rice heading under different nitrogen treatments. It is observed that with an increase in the nitrogen application, the heading date of rice with N300 is earlier by 1 day and heading duration becomes 1 day longer. This finding is consistent with the effects of nitrogen fertilizer on the timing of rice flowering [41]. The reliable and detailed phenotypic data provided by the proposed method can help us to discover agronomic phenomena dispensing with the laborious and time-consuming human observations.

### Flowering panicle identification and detailed trait analysis

The changing trend of the panicle number and the flowering panicle number with time series is shown in Fig. 8. The panicle numbers first increase rapidly and then tend to stabilize. The flowering panicle numbers first increase and then decrease, which is in line with the physiological changes of paddy rice. It is noted that in the 2019 dataset, the flowering panicle number on DAT 63 and DAT 64 is almost 0 as both of these days were rainy and cloudy, thus showing that the proposed classification model identified this sensitive change in rice flowering status under sudden environmental changes. In addition, it shows that when the flowering panicle number reaches the peak, the panicle number also enters a period of slow growth, which is very helpful for us to understand the synchronous relationship between the heading and flowering of rice. The closer the rising curves are, the more synchronized is the heading and flowering. Otherwise, there is a time lag between heading and flowering, which provides a new method for us to evaluate the performance of heading and flowering under different varieties and treatments. By comparing the fn curves under different nitrogen treatments, the consistency of the trend reflects that the proposed model has good adaptability in terms of flowering panicle identification under different treatments. Secondly, by calculating the flowering duration of the rice group, it shows that the increase in nitrogen fertilizer prolongs the flowering duration of the rice group, which is also similar to the results presented in Ref. [42], indicating that the proposed method is reliable in flowering panicle analysis.

The flowering time traits of panicles refer to the opening and closing time of panicles in each day after rice heading, during one of the most important periods in its life. The spikelet flowering of rice is completed in an instant, and it is relatively difficult to accurately record each flowering moment. Currently, research regarding flowering time is generally for the group. In this work, the detected panicles are classified into vigorous flowering and non-vigorous flowering, and the daily change curve of the maximum vigorous flowering panicle number is drawn. We consider the time when the flowering panicle number reaches the peak of a significant increase as the vigorous flowering time for the rice group. This is timelier and more reliable as compared to human visual inspection [4,43]. Table 4 reflects the difference between the proposed method and manual observations from images. Most of the time, the difference is less than 10 min under different treatments, which is exactly the average time of field survey for our plot. In traditional investigation, people need to shuttle in the paddy fields and mark the opening spikelet, which touches the flowering panicles, making a big impact on researches such as artificial pollination, observation of anthers, and stigma exposure. On the contrary, our method non-destructively observes the flowering dynamics of rice in the field and tries to record the natural status. This not only avoids tedious and time-consuming manual inspections but also helps us to accurately record the flowering characteristics of rice panicles.

In addition, the daily changes in flowering panicle number and the cumulative flowering panicle number under different nitrogen treatments are analyzed. The effect of nitrogen treatments from the perspective of the rice group is observed, which leads to a new question, i.e., how does the individual rice panicle flowering perform? Is the performance similar to that of the rice group or not? Most of the current researches focus on anther segmentation of flowering panicles, pollen viability identification, or spikelet counting. The detailed flowering phenotypes are obtained by panicle tracking in this research, and it helps us to discover how nitrogen affects the individual panicles. This is rarely performed in previous research.

We completed the tracking of individual panicles within each day during the heading–flowering stage, and the performance can be considered efficient. The tracking applied on images across days failed because the panicles’ tracking IDs change a lot between 2 adjacent days (i.e., 18:00 on the previous day and 8:00 on the day itself). It was caused by the lack of data during the night, which leads to relatively large displacement of panicle in images between 2 adjacent days. Based on the individual panicle location tracking on each day, we calculated FDAT, FDs, VFT_p_, daily DBE, and total DBE. As presented in Table 5, it reflects the nitrogen effects on individual panicle flowering. Combined with the effects on the rice group, we find that the increase in nitrogen application increases flowering panicle accumulation number, not only by earlier flowering and delayed ending both in groups and in individuals (reflected on FDAT and FDs), but also by the increase in the total DBE of each panicle. In addition, it is noted that rice flowering occurred earlier with larger nitrogen application in the 2020 experiment, which is different from the work presented in Ref. [42]. However, Zhang et al. [41] found that some varieties show a similar response; that is, their flowering day is brought forward by high nitrogen and delayed by low nitrogen.

### Prospects

Multi-task learning (MTL) is a powerful technique that enables a single model to perform multiple related tasks simultaneously. MTL offers several advantages, including improved model efficiency and generalization performance, since shared features learned across tasks can enhance the performance on all tasks [44]. However, MTL requires careful balancing of tasks during training and careful selection of tasks that are sufficiently related to benefit from shared feature learning. In this research, we chose to complete the individual tasks separately to increase the flexibility of the approach and ensure extension. However, in the future, we plan to integrate this process and build a model suitable for multi-tasking, once a reasonable and reliable approach was established. This will allow us to leverage the benefits of MTL and further improve the model performance.

In this research, since we only analyzed one variety of rice in the 2020 experiment, the current results may not be fully consistent with previous studies. The effects need to be discovered on more different types of rice varieties, e.g., indica, japonica, and further subdividing into conventional rice, hybrid rice, super hybrid rice, etc. Additionally, rice flowering is greatly influenced by environmental factors [45]. In the future, the experiment will be successively performed with more varieties, to verify the effects through years of experiments. However, in general, the proposed method can obtain more phenotypic characteristics of rice panicles at the heading–flowering stage, providing a new way of analysis for agronomists.

The nitrogen effects on rice heading and flowering, may help us avoid extreme weather and achieve a stable production. However, it also changes the start of grain filling. It is well known that the process of grain filling is essential to the formation of rice yield. Although increasing the nitrogen application increases the panicle number, its effects on grain filling initiation and grain filling duration cannot be ignored. Therefore, balancing the relationship between panicle number, flowering, grain filling, and nitrogen application, then choosing the appropriate nitrogen application rate to maximize the yield is the direction we need to study in the future.

Moreover, there is a difference between superior and inferior grains on the panicle. The superior grains are located in the upper part of the panicle, while the inferior grains are located in the lower part of the panicle. Compared to the superior grains, the inferior grains flower later and have a slower grain filling initiation, a lower filling rate, and a lower final grain weight [46]. This difference ultimately leads to a high or low yield and differences in quality of the 2 parts. Previous studies show that nitrogen fertilizer has a great impact on rice grain filling [47,48]. In order to further investigate the effect of nitrogen fertilizer on panicle development, we will classify panicles according to the flowering positions on panicle, quantify flowering process, and determine the start time of grain filling in different panicle parts, so as to provide more reliable and helpful analysis for grain filling between different panicle shape types.

## Conclusion

In this work, we used the YOLO v5, ResNet50, and DeepSORT models to perform rice panicle detection, flowering panicle identification, and identical panicle tracking. We proposed a pipeline to realize not only the panicle counting, heading date identification, and vigorous flowering time observation for the rice population, but also the detailed flowering phenotypic parameters estimation for a single panicle. The proposed method is non-destructive, accurate, and fast; is a time-series approach; and helps the researchers to easily obtain the panicle traits. Furthermore, by analyzing rice panicle phenotypes under different nitrogen treatments, the effect of nitrogen application on panicle development during the heading–flowering stage was explored. In future work, we will further use MTL to improve the model performance, verify the effects with more rice varieties and environmental factors, discover the flowering change of positions in panicle, quantify the degree of flowering, and extend this work to grain filling and maturity stages.

## Data Availability

The models used in this study are available in Github (https://github.com/Kyangzhou/data). The datasets analyzed in this study are available from the corresponding author upon reasonable request.

## Supplementary Materials

Supplementary 1 Supplementary Material 1. Fig. S1. Training results of the panicle detect model based on YOLOv5s6. Fig. S2. Scatter distributions of daily flowering panicle number under different nitrogen treatments.

Supplementary 2 Supplementary Material 2. Example videos of tracking results under different nitrogen applications in 2 years in DAT 67.
