# Supplementary material for: Analyzing Nitrogen Effects on Rice Panicle Development by Panicle Detection and Time-Series Tracking
Source: Plant Phenomics. 2023 Jun 23;5:0048. doi: 10.34133/plantphenomics.0048 (PMC10289797; doi:10.34133/plantphenomics.0048)
Supplement: Supplementary 1 — Supplementary Material 1. Fig. S1. Training results of the panicle detect model based on YOLOv5s6. Fig. S2. Scatter distributions of daily flowering panicle number under different nitrogen treatments. [file plantphenomics.0048.f1.docx]

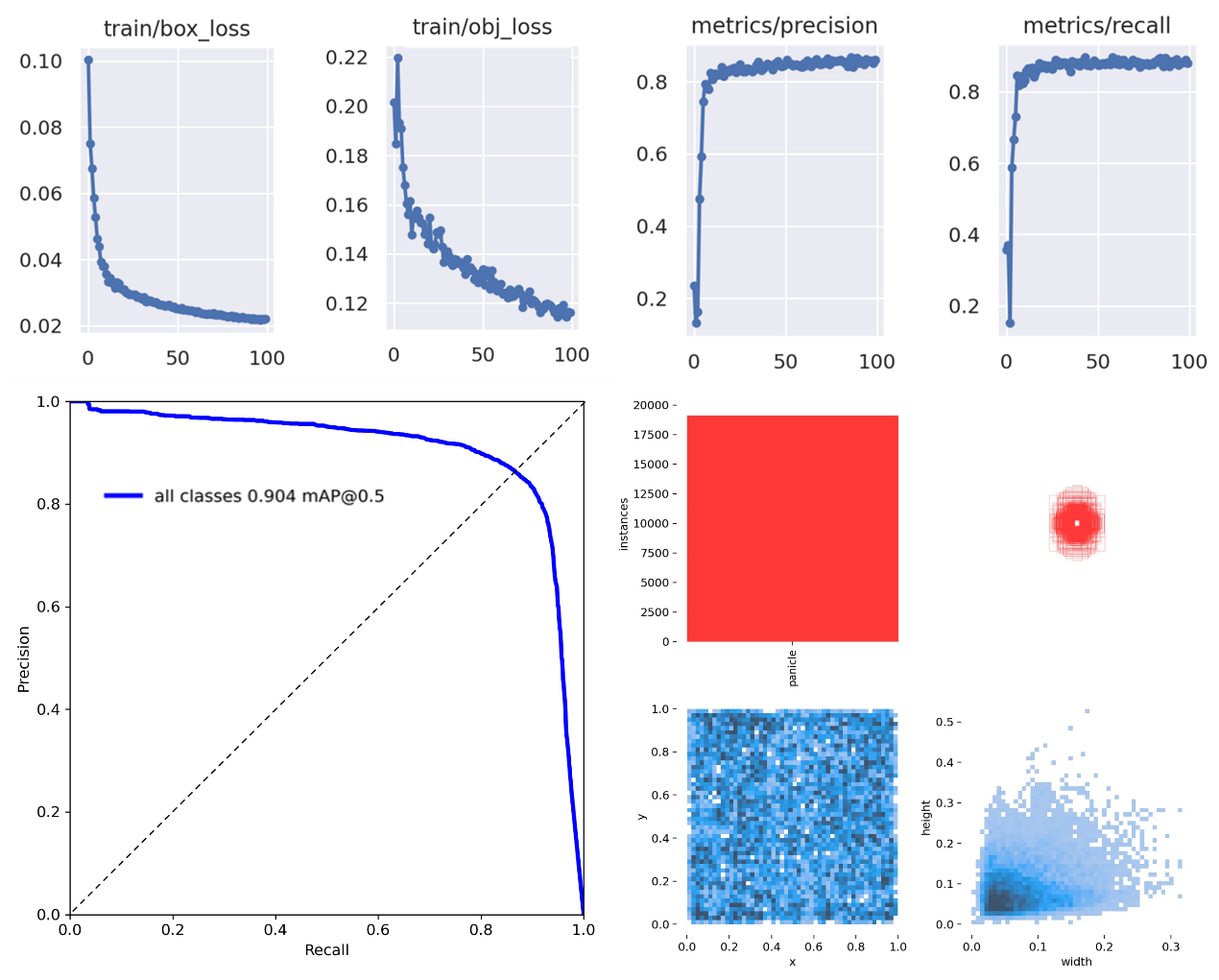


**Figure S1** Training results including box loss, object loss, precision, recall, the PR_curve and labels information including: amount of data per category, label shape, position of the label’s center, the length and width of labels.


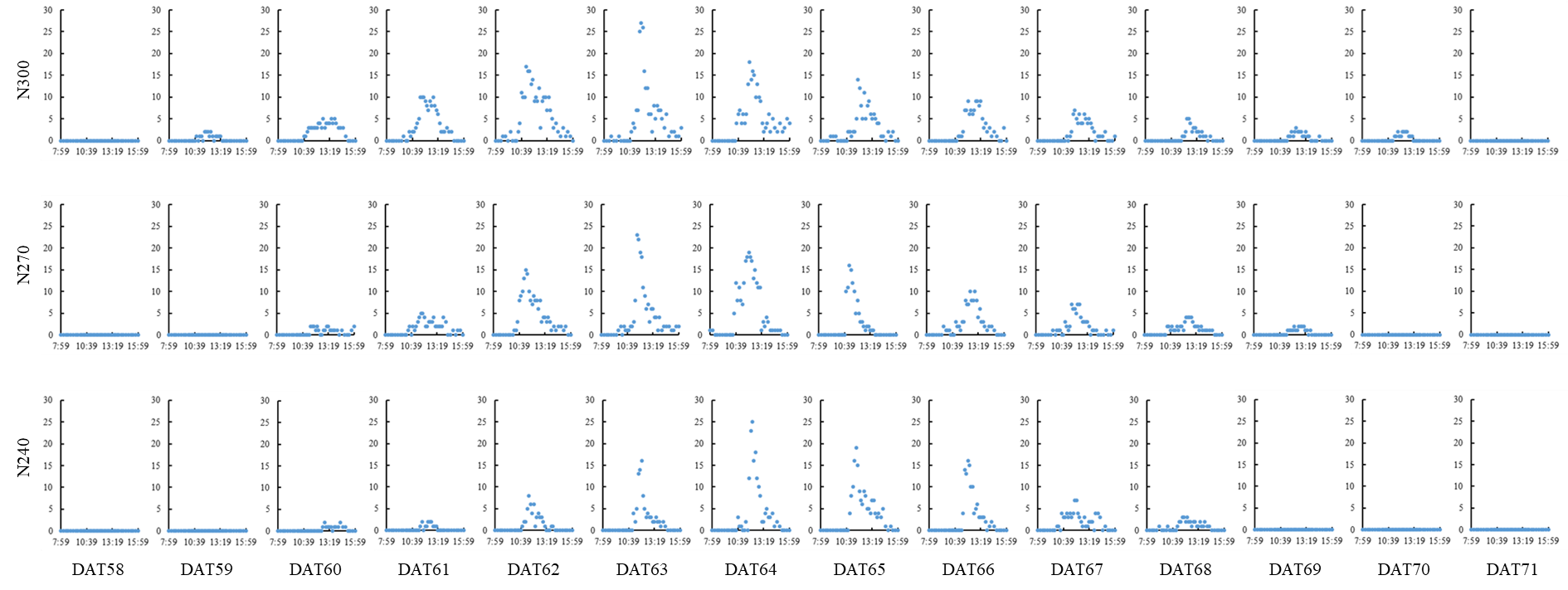


**Figure S2.** Scatter distributions of daily flowering panicle number under different nitrogen treatments.
