# Supplementary material for: Analyzing Nitrogen Effects on Rice Panicle Development by Panicle Detection and Time-Series Tracking
Source: Plant Phenomics. 2023 Jun 23;5:0048. doi: 10.34133/plantphenomics.0048 (PMC10289797; doi:10.34133/plantphenomics.0048)
Supplement: Supplementary 2 — Supplementary Material 2. Example videos of tracking results under different nitrogen applications in 2 years in DAT 67. [file plantphenomics.0048.f2.docx]

**Note: Double click on the icon to activate the videos.**
